# Feet deformities and their close association with postural stability deficits in children aged 10–15 years

**DOI:** 10.1186/s12891-019-2923-3

**Published:** 2019-11-13

**Authors:** Beata Szczepanowska-Wolowiec, Paulina Sztandera, Ireneusz Kotela, Marek Zak

**Affiliations:** 1 Faculty of Medicine and Health Sciences, Institute of Physiotherapy,The Jan Kochanowski University, Al. IX Wiekow Kielc 19, 25-317 Kielce, Poland; 2 Rehabilitation Clinic, Provincial General Hospital in Kielce, ul, Kościuszki 3, 25-310 Kielce, Poland; 3 Central Clinical Hospital of the MSWiA in Warsaw, ul, Wołoska 137, 02-507 Warsaw, Poland

**Keywords:** Public health, Feet deformities, Balance, Postural stability, Children

## Abstract

**Background:**

Children and young people make up an age group most vulnerable to falls. Various stability disorders may become instrumental in sustaining more frequent falls and resultant fractures. Correct morphological structure impacts overall efficiency of the foot, as well as offers significant diagnostic potential. Even minor foot disorders may affect the entire bio kinematic chain, also impacting the foot’s motility. Structural alterations within a foot may also impair balance in the standing position, and contribute to more frequent injuries. The study aimed to assess the relationship between feet deformities and postural stability deficits in schoolchildren prone to sustain accidental falls.

**Methods:**

The study involved 200 children (101 girls and 99 boys) aged 10–15 years,

randomly selected from primary schools. A 2D podoscan was used to assess the plantar part of the foot, while stabilometric examination was aided by the FreeMed dynamometric platform.

**Results:**

Correlation between respective variables was reflected by Spearman’s rank coefficient. The subjects’ age negatively correlated with the COP range of movement along the Y axis, and the COP surface area, while their BMI negatively correlated with the COP trajectory’s length. Step regression analysis indicated that the width of the left foot, the left foot Wejsflog index, the left foot Clark’s angle, the hallux valgus angle were the essential predictors of stabilometric variables in girls. In boys, though, predictive value was associated with Clarke’s angle of the left and right foot, Wejsflog index of the right foot, and the width of both the left and right foot.

**Conclusions:**

There is a statistically significant correlation between morphological variables of the foot and postural stability. When assessing the key variables of the foot and their interrelationship with postural stability, the Clarke’s angle, Wejsflog index, hallux valgus angle, and foot width, should be granted due prominence in the girls. As regards the boys, the following variables were established as predictive in assessing postural stability: Clarke’s angle, Wejsflog index, and foot width.

## Background

Frequent injuries are regarded as an important health, economic, and social issue among children and adolescents, attracting attention of researchers from all over the world. Most commonly, injuries among children are caused by accidental falls, which also stand for the main cause of hospitalization in emergency wards in the United States [1]. Hedstrőm et al. [2], while examining Swedish children, established that approx. 30% of them sustained at least one fracture. The investigators focused on the issue of balance disorders as a potential causative factor. They also managed to establish that it was mainly the boys (61% of the study population) who were more likely to sustain accidental fractures [2].

This assertion was also corroborated by others, e.g. Halawa et al. [3], Heidenkeni et al. [4], Ndung’u et al. [5]. Overall assessment of morphological structure of the feet, and any attendant deformities in terms of their impact on postural stability, is therefore believed an essential and challenging research issue in many areas. Correctly developed morphological structure affects overall efficiency of the feet, while also boasting significant diagnostic potential. Even minor dysfunctions within a foot may consequently impact the entire bio kinematic chain, consequently affecting its motility. Dysfunctions of the loco motor system, though, may cause lower limb failure, translating into degenerative changes in the peripheral and spinal joints. Structural alterations within a foot may also contribute to impaired balance in the standing position [6–10]. As evidenced by our own research, foot deformities are bound to impact overall postural stability.

Principally, postural stability relies on a properly developed bodily structure, i.e. overall efficiency of nervous, osteoarticular, ligamentous, and muscular systems. Overall complexity of the balance maintaining process becomes apparent only when postural disorders, pathological, or involutionary changes occur within the body. Then, postural stability gets disturbed as a natural consequence [11–13]. In statics, assessment of an individual sense of balance is carried out by assessing the shifts of a bodily centre of gravity (COG), while maintaining a still stance. These postural sways can be assessed with the aid of special stabilometric platforms recording the pressure of the feet against their surface, whereupon pressure and torque are recorded by the sensors embedded within. Based on these readings, the position of the centre of foot pressure (COP) is calculated. In static conditions, not only does COP show the deflection of COG, but also the force of foot pressure against the platform itself. Measuring the COP signal with the aid of a stabilometric platform is an objective method of assessing individual balance effectively [8, 12].

Numerous investigators focused on the feet as the principal subject of their research, while trying to gain some insights into how their morphological structure may actually affect overall efficiency of an individual loco motor system, and a sense balance at large [14–18].

There are very few studies focused on the impact of the foot structure on postural stability in children and adolescents, which prompted the present Authors to pursue their own research into the issue.

The study aimed to assess the relationship between feet deformities and postural stability deficits in schoolchildren prone to sustaining accidental falls.

## Methods

### Participants

The survey involved 200 children (101 girls and 99 boys) aged 10–15 years, randomly selected from primary schools, representing both the urban and rural environment of a single province, whose characteristics are presented in Table 1. All study subjects had been granted permission to attend by their parents/guardians.

**Table 1:** Basic characteristics of the study group

Variable	Boys (*n* = 99) Mean ± SD	Girls (*n* = 101) Mean ± SD	Z	*p*
Body mass [kg]	47.56 ± 11.99	44.92 ± 11.46	− 1.285	0.199
Height [cm]	154 ± 0.12	153 ± 0.11	−0.35	0.727
BMI	19.78 ± 3.28	18.91 ± 3.22	−1.853	0.064

Mean-arithmetic mean, SD-standard deviation, Z- statistical values of the Mann-Whitney-Wilcoxon test for two independent samples, BMI- body mass index, p-significance level

Table 1 comprises basic somatic characteristics of the study group.

### Design

The study protocol was pursued in the Posturology Laboratory. Body mass was measured using Tanita scales (manufactured in Japan 93/42/EEC Annex II; measurement accuracy ±0.1 kg), while the height with the aid of SECA stadiometer (manufactured in Germany 93/42/EWG, 2007/47/WE; measurement accuracy 0.01 m).

**Fig. 1:**
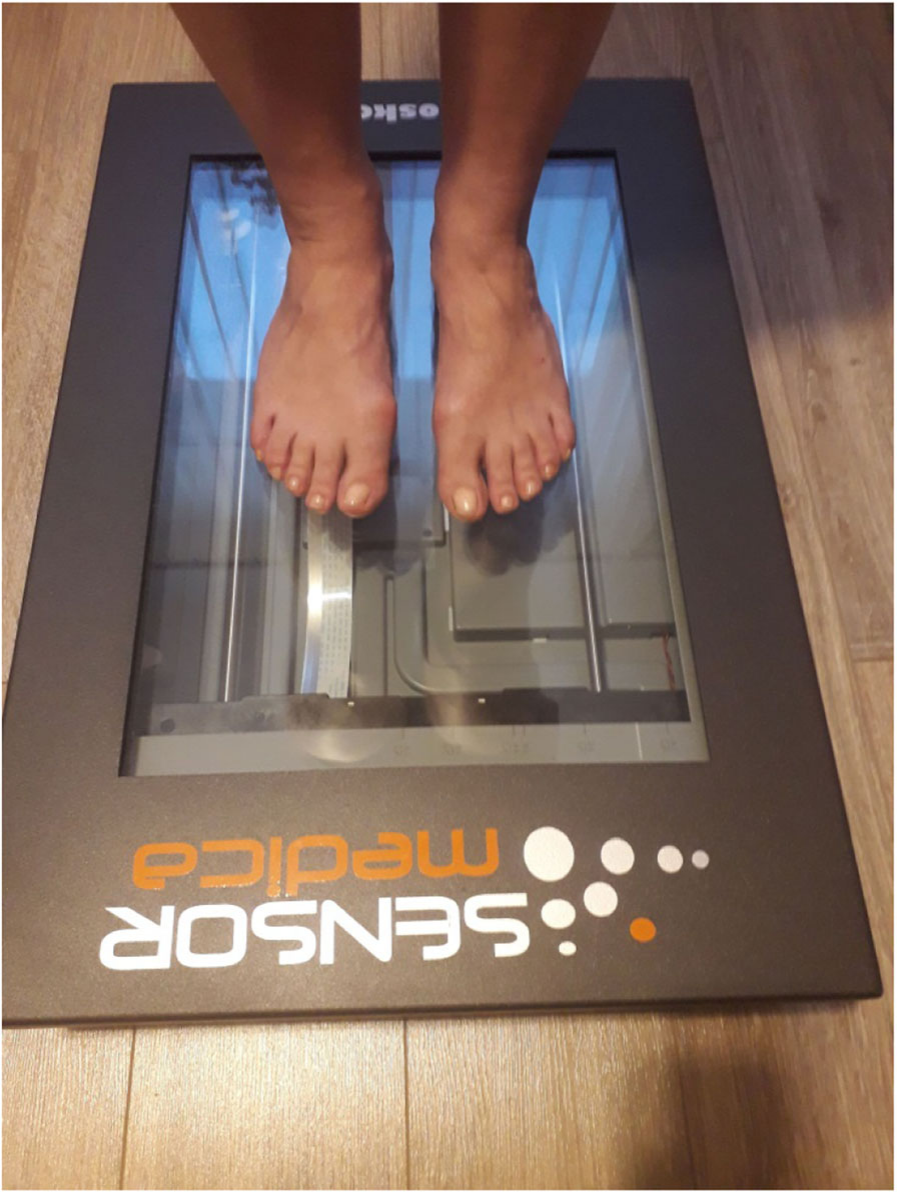
PodoScan 2D. Source: own research materials

The assessment of the plantar part of the foot in static conditions was pursued using an Italian-made PodoScan 2D FootCAD, fitted with a CCD converter with a cold cathode (1600DPi). This technologically advanced device facilitates digital analysis of the plantar footprints and loads. The actual image of the plantar part of the foot is analysed, so that its length, width, angles and axes are determined. During the test, the subjects stood on the device barefoot, the lower limbs upright, the upper limbs hanging along the body, the feet parallel. Examination of the feet was carried out under the load of their own weight. (Fig. 1).

**Fig. 2:**
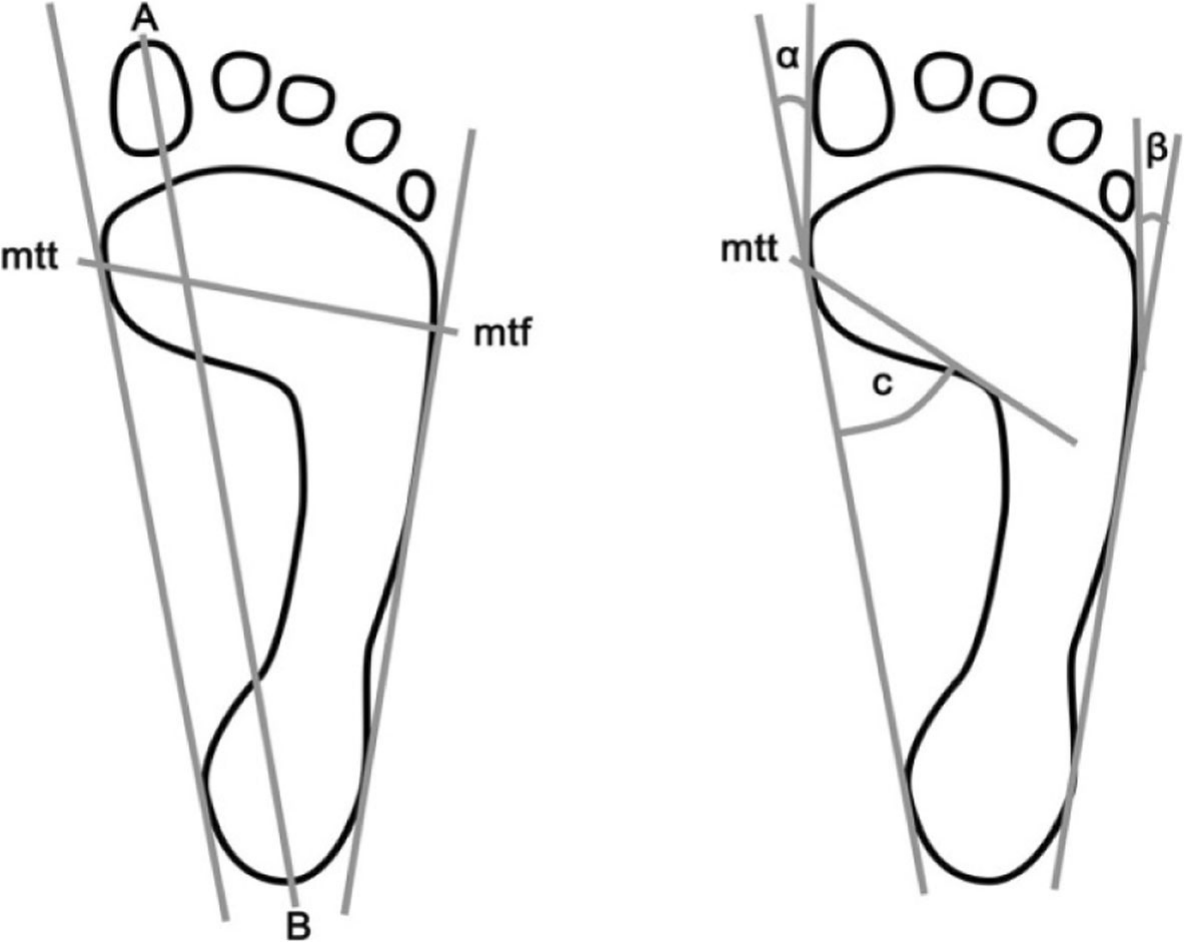
The method of determining the foot indicators under study. **a-b**-length of the foot, mtt-mtf-width of the foot, **c**-Clarke’s angle, α-hallux valgus angle, β- angle of deformity of the fifth toe. Source: own research materials

The following indicators were assessed, foot length - the line connecting the furthest points of the forefoot and hindfoot - in mm, forefoot width - the line joining the most extreme points on the head of the first (mtt) and the fifth metatarsal bone (mtf) - in mm, Clarke’s angle - the angle between the tangent of the medial edge of the foot and the line joining the point of the largest indentation and contact of the medial tangent with the foot edge - in °, the Wejsflog (W) index - length-to-width ratio, the hallux valgus angle (α) – the angle between the tangent to the medial edge of the foot and the tangent of the edge of the hallux, derived from the mtt point - in °, The angle of the varus deformity of the fifth toe (β) - the angle between the tangent of the lateral edge of the foot and the tangent of the edge of the fifth toe derived from the point mtf - in ° (Fig. 2) [7, 8].

The longitudinal arch was assessed on the basis of the Clarke’s angle value, assuming that a flat foot is construed within the > 30° range, a foot with a reduced arch - 31° - 41° range, a correctly arched foot - 42° - 54° range, and a foot with a raised arch - < 55°. Whilst taking into account the Clarke’s angle values we were able to determine whether the foot under study was flat, correctly structured, or whether it was a cavusfoot [19]. The transverse arching was evaluated using the Wejsflog index (length/width of the foot, ratio 3:1). The values closer to “2” indicated a transverse flat foot, while the ones closer to “3” indicated its correct transverse vaulting.The hallux valgus angle (α), whose normal value is up to 9°, was also assessed.For the angle of the varus of the fifth toe, the mean values ​​of this angle were calculated As there are no published standards available for assessing the angle of the varus of the fifth toe, the mean values ​​of this angle were calculated.

**Fig. 3:**
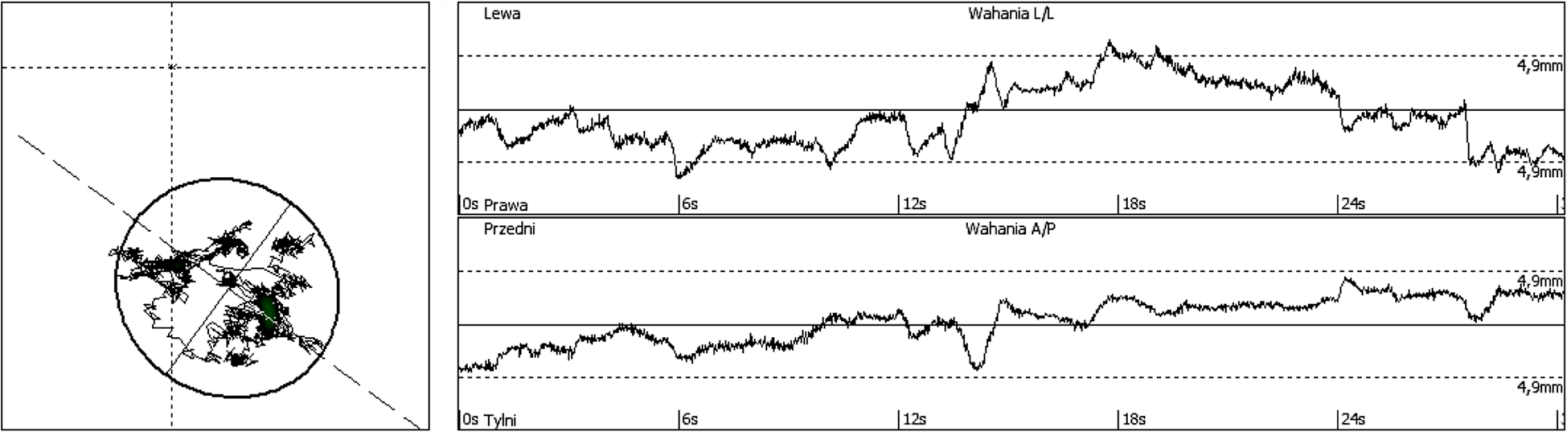
Examples of surface area and COP path charts. Source: own research materials. An sample of the study charts is comprised in Fig. 3. - length of sway: determines the length of the COP trajectory in mm – COP length. - surface: surface area of the COP sway in mm² - COP field area. - mean X: mean value in mm of the COP trajectory in the X-axis - COP X mean. - mean Y: mean value in mm of the COP trajectory in the Y-axis - COP Y mean. - X axis: the range of COP movement in the X axis in mm in the mid-lateral direction of ML - COP X. - Y axis: the range of COP movement in the Y axis in mm in the antero-posterior direction AP - COP Y

For girls: left foot mean-14.45 ± SD 5.53, right foot mean-13.86 ± SD 5.26 For boys: left foot mean-14.56 ± SD 5.15, right foot mean-15.17 ± SD 5.11.A stabilometric test was completed using a dynamometer platform (FreeMed, Sensor Medica, manufactured in Italy), operated by FreeStep Pro software n. 134 L-2010. The total surface of this platform is 635 mm × 700 mm, the active surface of the sensors - 500 mm × 600 mm. Sampling frequency is 300–350 Hz in real time. The platform facilitates the study of balance, and any disturbances in visual and motor coordination, as well as allows to assess the actual distribution of foot pressure [20].During the measurements, the subjects were standing in a relaxed position, their feet parallel, the upper limbs hanging along the body. The respondents were asked not to move and to look straight ahead. The measurement protocol lasted 30 s.The displacement of the Center of Foot Pressure (COP) was analyzed by assessing the following indicators, length of sway: determines the length of the COP trajectory in mm – COP length, surface: surface area of the COP sway in mm^2^ - COP field area, mean X: mean value in mm of the COP trajectory in the X-axis - COP X mean, mean Y: mean value in mm of the COP trajectory in the Y-axis - COP Y mean, X axis: the range of COP movement in the X axis in mm in the mid-lateral direction of ML - COP X, Y axis: the range of COP movement in the Y axis in mm in the antero-posterior direction AP - COP Y A sample of the study charts is comprised in Fig. 3.

### Statistical methods

The test results were statistically analysed using the R.3.5.0 programme. The basic metrics of descriptive statistics were used to characterize the material under study, i.e. arithmetic mean, standard deviation. The parameters of the structure of the right and left foot, in due consideration of gender, were analysed. The Mann-Whitney-Wilcoxon test for two independent assessments was used to assess the dependence of the variables under study on gender. When analysing the variables pertaining to the key structural features of the feet, though, Spearman’s rank correlation was applied. In order to verify the impact of the foot variables on posturographic parameters, progressive regression was used. The dependent variable was subjected to the Shapiro-Wilk test for the normality of distribution. When the test indicated that the variation did not have a normal distribution, Box-Cox transformation was applied. The quality of the model was also evaluated using the determination coefficient (R^2^). Individual variables were considered statistically significant, and consequently included in the model, if the F statistic from the Fisher-Snedecor test was *p* < 0.05.

The resultant dependencies were deemed statistically significant, if the level of significance was *p* < 0.05, when *p* < 0.01 - the dependence was highly statistical, and when *p* < 0.001 - the dependence was very highly significant statistically.

## Results

Table 2 presents a statistically significant relationship between the length and width of the left and right foot, and gender of the study subjects.

**Table 2:** Statistically significant morphological variables of the feet within the study group, stratified by gender

Variables	Boys		Girls		Z	*p*
	Mean	SD	Mean	SD		
Length - L foot	238.15	17.85	229.53	14.04	−3.094	0.002
Length - R foot	237.81	17.48	229.41	14.1	−3.118	0.002
Width - L foot	88.77	6.34	85.47	6.35	−3.399	0.001
Width - R foot	89.12	6.35	85.9	6.59	−3.128	0.002
Hallux valgus angle (α) P	4.16	3.48	6.12	4.1	3.642	< 0.001

Mean-arithmetic mean, SD-standard deviation, Z- statistical values of the Mann-Whitney-Wilcoxon test for two independent samples, p-level of significance, L-left, R-right

In Table 3, Spearman’s rank correlation was applied to determine the correlation between respective variables. The length of the right foot negatively correlated with the length of the COP trajectory and the range of COP along the Y axis. The width of the left and right foot negatively correlated with the length of COP sway, COP surface area; the range of COP movement along the Y axis and in the X axis.

**Table 3:** Correlations between the structural characteristics of the feet and the stability indicators within the entire study group

Variable	COP length	COP Surface area	COP Y	COP X
Length L foot	ρ = −0.126 *p* = 0.076	*ρ* = −0.114 *p* = 0.108	*ρ* = −0.134 *p* = 0.059	*ρ* = − 0.111 *p* = 0.119
Length R foot	*ρ* = −0.154 *p* = 0.030	*ρ* = − 0.124 *p* = 0.081	*ρ* = − 0.140 *p* = 0.049	*ρ* = − 0.125 *p* = 0.078
Width L foot	ρ = − 0.301 *p* < 0.001	*ρ* = − 0.173 *p* = 0.015	*ρ* = − 0.179 *p* = 0.011	*ρ* = − 0.174 *p* = 0.014
Width R foot	ρ = − 0.288 *p* < 0.001	*ρ* = − 0.160 *p* = 0.024	*ρ* = −0.171 *p* = 0.016	*ρ* = − 0.160 *p* = 0.024
Clarke’s angle R	*ρ* = 0.211 *p* = 0.003	*ρ* = −0.143 *p* = 0.043	*ρ* = −0.122 *p* = 0.084	*ρ* = − 0.152 *p* = 0.031
Wejsflog index L	*ρ* = 0.323 *p* < 0.001	*ρ* = 0.140 *p* = 0.048	*ρ* = 0.109 *p* = 0.125	*ρ* = 0.148 *p* = 0.037
Wejsflog index R	*ρ* = 0.281 *p* < 0.001	*ρ* = 0.077 *p* = 0.277	*ρ* = 0.061 *p* = 0.392	*ρ* = 0.078 *p* = 0.270
Hallux valgus angle α L	*ρ* = −0.078 *p* = 0.271	*ρ* = 0.028 *p* = 0.692	*ρ* = 0.000 *p* = 0.995	*ρ* = 0.038 *p* = 0.595
Hallux valgus angle α R	*ρ* = −0.092 *p* = 0.196	*ρ* = −0.115 *p* = 0.106	*ρ* = − 0.147 *p* = 0.038	*ρ* = − 0.091 *p* = 0.201
The fifth toe angle β L	*ρ* = − 0.193 *p* = 0.006	*ρ* = − 0.147 *p* = 0.038	*ρ* = − 0.146 *p* = 0.039	*ρ* = − 0.141 *p* = 0.046
The fifth toe angle β R	*ρ* = − 0.134 *p* = 0.059	*ρ* = − 0.181 *p* = 0.010	*ρ* = − 0.156 *p* = 0.028	*ρ* = −0.180 *p* = 0.011

ρ- Spearman’s rank correlation

The Clarke’s angle of the right foot positively correlated with the length of COP sways, even though it negatively correlated with the surface area of the COP and the range of COP movement along the X axis. The Wejsflog angle of the left foot and the right foot positively correlated with the length of COP trajectory. The Wejsflog angle of the left foot positively correlated with the surface area of the COP and the range of movement along the X axis. A negative correlation was observed between the hallux valgus of the right foot and the COP range on the Y axis. The angle of the fifth toe of the left and right foot correlated negatively with the COP surface area; the scope of COP movement along the Y axis; and range of COP movement along the X axis.

A negative correlation was found between the angle of the fifth toe of the left foot and the length of the COP movement.

In order to study the relationship between stability and gender, the Mann-Whitney-Wilcoxon test was applied for two independent assessments. A statistically significant correlation was established between the COP surface area and gender (Table 4).

**Table 4:** Relationships between postural stability and gender

Variable	Mean		SD		*p*	Mean	SD
	Boys	Girls	Boys	Girls			
COP length	710.54	730.78	245.63	236.05	0.47	720.76	240.44
COP surface area	227.86	105.05	799.85	193.36	0.029	165.84	581.07
Mean X	−4.52	−2.62	6.96	5.59	0.063	−3.56	6.36
Mean Y	−16.63	−15.56	9.64	8.73	0.265	−16.09	9.18
COP Y	15.17	11.18	26.79	8.8	0.062	13.16	19.91
COP X	10.97	8.64	7.62	5.1	0.023	9.79	6.56

In the group of girls, the Wejsflog index of the left foot was the only important predictor, accounting for 11% of COP length variance. 7.1% of the variance of the COP surface area accounted for by the width of the left foot; 6.6% of variability for the mean COP value in the X axis corresponded to the Clarke’s angle of the left foot; 6.6% of variability for the mean value of the COP in the Y axis corresponded to the valgus angle of the left hallux; 7% of variance of COP X accounted for the width of the left forefoot, whereas in the case of the COP Y, 11% of the variance corresponded to the width of the left foot and the valgus angle of the right hallux.

As regards the boys, the variance of the COP length in 23.4% was accounted for by taking into consideration the Wejsflog index of the right foot, the left foot width, and the Clarke angle of the right foot. 10.5% of the variance of the COP surface area accounted for the width of the right foot, and the Clarke’s angle of the right foot; 4.8% of variability for the mean value of COP along the X axis corresponded to the Clarke’s angle of the left foot; 19.6% of the variability for the mean value of the COP along the Y axis corresponded to the width of the right foot. In the case of the COP Y, 11.8% of variability was accounted for by the width of the right foot, and the Clarke’s angle of the right foot (Table 5).

**Table 5:** Statistically significant stepwise regression models

Gender	Dependent variable	Predictor	F	*p*	R^2^
Girls	COP-log length	Wejsflog index L	12.14	0.001	0.109
	COP-log Surface area	Width L foot	7.55	0.007	0.071
	X Mean	Clarke’s angle L	6.94	0.010	0.066
	Y Mean	Hallux valgus angle L	6.97	0.010	0.066
	COP Y-log	Width L foot	6.98	0.010	0.110
		Hallux valgus angle R	5.11	0.026	
	COP X-log	Width L foot	7.40	0.008	0.070
Boys	COP-log length	Wejsflog index R	16.51	< 0.001	0.234
		Width L foot	8.15	0.005	
		Clarke’s angle R	4.34	0.040	
	COP-log surface area	Width P foot	4.93	0.029	0.105
		Clarke’s angle R	6.36	0.013	
	X mean	Clarke’s angle L	4.88	0.030	0.048
	Y mean	Width R foot	9.62	0.003	0.196
	COP Y-log	Width R foot	5.77	0.018	0.118
		Clarke’s angle R	7.04	0.009	

F- statistical values, Fisher-Snedecor test, R^2^ - determination coefficient, log - logarithm of the variable

## Discussion

The nature of the associations encountered between the key structural variables of the foot and overall postural stability in adults has long attracted wide academic interest. There are very few studies, however, focused upon the actual impact of specific structural variables of the foot on postural stability in children [2, 3]. The results of applying the stepwise regression model made it possible to identify the key variables within the structure of the foot which are believed to account for the specific values of the stability indicators under study.

Whilst assessing morphological structure of the feet in our own studies, the higher values of both the length and width of the feet among the boys rather than among the girls were noted. The width of the feet in the group under study significantly affected the COP surface area, very much like in the studies pursued by Xu et al. and Angin et al. [21, 22].

The values of Clarke’s angle and Wejsflog index remained well within the reference values range, both with regard to the left and right foot. Higher values of Clarke’s angle and Wejsflog index were observed in the girls, though, which is generally accounted for by sexual dimorphism in children. The values of the hallux valgus angle (α) of the left and right foot also fell within a normal range, even though in the girls they proved higher. Similar correlations were established in the studies conducted by Puszczalowska-Lizis [23]. Altogether different correlations were observed by Brzezinski et al. [24] who noted more frequent incidence of lower limb conditions among the boys, including flat footedness.

Studies of young adults and elderly persons corroborate the association between the foot arches and postural stability. Kim et al. [25] observed that the rate of COP sways was higher in the flat footed persons than in those with the correctly arched feet. This correlation was apparent in the static conditions, whereas in the dynamic ones such correlations were not noted. Birinci et al. [26] asserted that the reduced mobility of the foot arch was bound to affect overall postural control and COP sways. These changes induce a different arrangement of the higher located segments of the body, as they simply have to adapt accordingly. Also Cote et al. [27] reported that the actual structure of the foot affected overall postural stability, be that in static or dynamic conditions. Tahmasebi et al. [28], and Chao et al. [29], when comparing a group of the flat footed patients with the ones with the correctly arched feet, observed that there was an appreciable deterioration of overall postural stability in the flat footed subjects.

Cobb et al. [30] highlighted in their studies the correlation between postural stability and the Clarke’s angle; this having also been corroborated by our own research. The investigators noted that the actual height of the foot arch contributed to reduced stability of the entire posture, as well as that reduced mobility of the cavusfoot may well be the causative factor. Increased vaulting of the foot (both longitudinal and transverse) affected the COP length, as also evidenced in our own investigations.

Correct morphological structure of the foot projects onto its overall efficiency and daily functioning. The front support zone is essential, as it significantly affects the correct positioning of the toes. Any alteration in the toe angle setting is consequently reflected in overall postural stability. The hallux valgus angle in the group of girls was a significant variable affecting the COP Y, and average Y variables. This implies that an increased angle of hallux valgus is bound to affect overall postural stability. This implication is well corroborated by our own research, being also deemed a correlation of statistical significance. The angle of the small toe correlated with the COP surface area.

Wrigh et al. [31] reported the impact of a correct foot structure on the key variables of postural stability. An essential component that stands for overall postural stability consists in the structure of the metatarsal bones, and the actual positioning of the toes. Cinar-Medeni et al. [32] noted that even a small toe valgus might well be instrumental in adversely affecting individual postural stability. The subjects characterised by a wider hallux valgus angle displayed appreciably worse postural stability. Drzał-Grabiec et al. [33] also drew attention to the association between hallux valgus and overall stability indicators; similar correlations having been established in our own studies.

While assessing the morphological structure of the foot, it is well-worth taking due note of its association with overall postural stability. Such associations are corroborated by other authors’ studies [21, 22, 25–33], as well as our own investigations. Being well aware that various foot abnormalities may well be instrumental in the deformation of other constituent components of the musculoskeletal system, gait, and overall postural stability, regular feet monitoring appears more than well justified as a routine healthcare practice. Any such monitoring practice, when pursued with the aid of modern, repeatable methods, might appreciably contribute to reliable, large scale population studies.

Precise analysis of the results allows to focus early enough on the emerging postural abnormalities, or those regarding individual balance, and consequently implement a target-oriented therapeutic management. The option of data archiving/retrieval makes it possible to have the results juxtaposed and compared, as well as have the physiotherapeutic methods already in use verified for their overall effectiveness. Overall therapeutic effectiveness is dependent upon effective identification of the mechanisms which actually control postural stability, and their correlation with any existing deficits in this particular area [34].

It would therefore seem highly advisable to make use of modern diagnostic equipment, prudently taking advantage of its appreciable potential. Admittedly, not only is effective interpretation of pertinent readouts of paramount significance for the actual diagnosis, but it also warrants overall effectiveness of any subsequent therapeutic management.

Further in-depth research into this issue is still required, allowing for more numerous study population, and focused primarily on the specific feet deformities, especially in terms of age stratification.

### Limitations of the study

An appreciable limitation of the present study consisted in pursuing the tests on the children with a low body weight.

## Conclusions

1. There is a statistically significant correlation between morphological variables of the foot and postural stability.

2. When assessing the key variables of the foot and their interrelationship with postural stability, the Clarke’s angle, Wejsflog index, hallux valgus angle, and foot width, should be granted due prominence in the girls.

3. As regards the boys, the following variables were established as predictive in assessing postural stability: Clarke’s angle, Wejsflog index, and foot width.

## Data Availability

The datasets generated during and/or analysed during the current study are available from the Corresponding Author on reasonable request.

## Abbreviations

COG: Centre of Gravity
COP: Centre of Foot Pressure
